# Work of Foreign Veterinarians in Tuberculosis1Read before the Section on Public Health of the New York Academy of Medicine, November 8, 1895.

**Published:** 1895-12

**Authors:** Leonard Pearson

**Affiliations:** Veterinary Department of the University of Pennsylvania


### WORK OF FOREIGN VETERINARIANS IN TUBERCULOSIS.[1]

By DR. LEONARD PEARSON,
VETERINARY DEPARTMENT OF THE UNIVERSITY OF PENNSYLVANIA.

In the short time that I shall occupy I shall express no views of my own, but shall call your attention to some of the recently expressed opinions of European veterinarians on the subject of bovine tuberculosis and some European practices and rules in relation to this matter.

[1] Read before the Section on Public Health of the New York Academy of Medicine, November 8, 1895.

This subject has been a live one in Europe for many years, and has received much attention ever since it was shown by Villemin, in 1868, that the disease could be transmitted from one animal to another, and more especially since the discovery of the tubercle bacillus by Koch, in 1882, and the consequent establishment of the fact that tuberculosis of man and the lower animals is the same disease and caused by the same germ.

The first practical application of this knowledge, with a view of preventing human infection through the ingestion of tuberculous food of animal origin, was in relation to the inspection of meats. Without tracing the development of official meat-inspection in the various countries in Europe, suffice it to say that most of the European countries have a more or less perfect system of meat-inspection which is carried out most carefully in the great centres of population, and usually insures the consumer against harmful flesh.

The method that is in vogue in many cities of Germany, and is being extended as rapidly as possible, consists in the erection of a municipal abattoir in which all local slaughtering must be done, and where the food-animals can be examined by the veterinarians stationed there, both before and after slaughter. This allows a perfect inspection, which is impossible when the killing is done in a great number of small slaughter-houses scattered throughout a large city.

The question as to what shall be done with tuberculous carcasses has excited much discussion. There is a great unanimity of opinion in reference to the carcasses of animals that show generalized tuberculosis or tuberculosis with marked emaciation; they should be destroyed outright. But cases of localized tuberculosis are much more common, amounting in some places to 15 per cent. or 18 per cent. of all cattle slaughtered, and to destroy these carcasses would occasion great loss, which should be avoided, if it is possible to do so without prejudice to human health. The careful experiments of Chauveau, Nocard, Bollinger, Bang, and McFadyean have shown that the flesh of animals with local tuberculosis is usually not infectious. But Ostertag and others call attention to the fact, which is brought out more clearly by the Royal Commission on Tuberculosis in its report of 1895, that healthy muscles may carry tuberculous lymph-glands between them, and that in dressing carcasses the butcher frequently gets tuberculous material on his knife, if such exists in any part of the body, and thus spreads it on to otherwise harmless meat. Hence, it has been evident for a long time that great care must be exercised in judging tuberculous carcasses. Since 1886 it has been the custom in some countries, as in Portugal, to destroy the carcasses of all tuberculous animals, and in Italy, Austria, France, Germany, and Belgium much flesh was formerly destroyed that is now saved. When meat-inspectors were few and the systems imperfect it was necessary, in order to be on the safe side, to have few and rigid rules; but with the increase of experience and knowledge it has been found that these rules can be modified and made flexible when they are enforced by skilled men. At the International Veterinary Congress held last September in Berne it was decided by resolution that the flesh of tuberculous animals should be condemned when the carcass is emaciated, when it has a general bad appearance, when tubercles are found in the muscular portions, and when alterations are found in several organs. It was also recommended, in relation to the flesh of slightly tuberculous animals permitted to go on the market, that it be sold in special shops or stalls, or that it be sterilized and sold as cooked meat. But this resolution implies a doubt as to the wholesomeness of some of the meat passed, and leads one to the conclusion that a fear exists that the rules for condemning are not sufficiently comprehensive. In Germany the practice is to condemn the worst cases, sterilize those that are less extensive, and to pass as sound the slightly developed cases, after destroying the affected parts.

A very important point in connection with this subject is in reference to the payment of indemnity to the owner of the condemned animal or carcass. The opinion has spread rapidly of late years, that where an animal is condemned for the good of the public, the public should bear part of the loss. That this view is established abroad is shown by another resolution adopted by the International Veterinary Congress to the effect that owners should be compensated for animals destroyed, and this is already the practice in France.

The consideration of the milk from tuberculous cows is another major division of this subject, and probably of greater importance to the sanitarian than their flesh, for, while the latter is usually eaten after cooking and more or less complete sterilization, the former is usually consumed raw.

Numerous investigations have demonstrated that the milk of cows with tuberculosis of the udder will cause tuberculosis in a very large percentage of the experimental animals fed upon it, and, in reference to such experiments, the Royal Commission on Tuberculosis reports in these words: "We cannot refuse to apply, and do not hesitate to apply, to the case of the human subject the evidence thus obtained from a variety of animals that differ widely in their habits of feeding—herbivora, carnivora, and omnivora. As regards man we must believe—and here we find ourselves agreeing with the majority of those who gave evidence before us—that any person who takes tuberculous matter into the body as food incurs some risk of acquiring tubercular disease. By tuberculous matter we mean here, of course, that which is capable of giving rise to tuberculosis in lower animals."

The active bacilli of tuberculosis have been found in the milk of tuberculous cows by numerous investigators, and among them such distinguished veterinarians as Chauveau, Nocard, Bollinger, and Johne. At the Seventh International Congress of Hygiene, held in London in 1891, Prof. Bang, of Copenhagen, reported his experiments with fifty-eight tuberculous cows, of which nine were found to give virulent milk. He expressed the opinion that a tuberculous cow with udder apparently healthy is not in the great majority of cases dangerous, although she is undoubtedly so sometimes, and is always suspicious. Professors McFadyean and Woodhead, who reported at the same time, demonstrated the extreme danger of cows with tuberculous udders and the occasional infectiousness of milk from cows with the disease in organs other than the udders. From the results obtained from the experiments, and by all other investigators, they conclude that the milk obtained from tuberculous animals may be instrumental in causing the disease in man, and that there is great necessity for legislation on this question. They state further that the first thing to be insisted on is that a regular staff of veterinary inspectors, well trained for this special work, shall be appointed, whose special duty it must be to examine fortnightly all the cows giving a milk-supply, and who should have the power to order isolation of all cattle in which the presence of tuberculosis is suspected.

In the more recent report of the Royal Commission on Tuberculosis in Animals the statement is made that the withdrawal from dairies of every cow that has any disease whatever of the udder would form some approach to security against the serious danger incurred by man "from the use of tuberculous milk," but it would not be an adequate security. "The presence in a dairy of a tuberculous cow," the report says, "is a decided source of danger to the public, especially having regard to what we have learned respectiug the rapid development of tuberculosis of the udder, and the degree of danger to milk-consumers incurred by the invasion of the udder in tuberculous cows." Attention is also called in this report to a most important observation in reference to the alarming rapidity with which tuberculosis of the udder may develop, and cases are cited in which this condition became evident between fortnightly inspections. The investigators for the Commission insist that no tuberculous animal of any kind should be allowed to remain in a dairy. Ostertag has called attention to the fact that to exclude from sale the milk of all cows that react to tuberculin would mean a great shrinkage in the milk-supply in some places, and would cause a great and oppressive rise in the price of this necessity. He recommends, therefore, that the milk from cows with tuberculosis of the udder should be excluded from consumption, and that from cows which react to tuberculin, but show no evidence of tuberculosis of the udder, should be sterilized before sale.

Another important division of the subject assigned to me is that in reference to the eradication of this disease from herds, and, since all exterminative measures necessitate the diagnosis of disease in the living animal, I shall present some views in relation to tuberculin as a diagnostic agent, for diagnosis without this agent is so inaccurate and untrustworthy, except in advanced cases, that it now receives but little credence. The use of tuberculin is so recent and its career as a therapeutic agent was so short and disappointing that there was a strong feeling against its employment as a diagnostic agent in cattle-practice until its worth and harmlessness had been demonstrated beyond a doubt. Nocard in France, Bang in Denmark, Guttmann in Russia, and Siedamgrotzky in Germany, have taken the lead in this work.

The original researches with tuberculin showed from 10 to 15 per cent. of failures to diagnose correctly; but with improved methods and increased experience the proportion of failures in the hands of careful veterinarians is now so small as to be almost disregarded. Nocard, Guttman, and Bang, who have had the most experience with this agent, claim almost invariably exact and accurate results. There was a tendency among a few veterinarins at the recent International Veterinary Congress, and notably on the part of Guillebeau and Hess, of Berne, to oppose the use of tuberculin for diagnostic purposes on the ground that it caused preëxisting tubercular processes to become generalized. This view, however, was not supported by any of the other veterinarians present, and was expressly refuted in the resolutions adopted. These resolutions, which represent the most recent opinions of the foremost veterinarians in Europe, and which were adopted at the International Veterinary Congress held but two months ago, are of great interest to us, for they are based on five years of trial, experiment, and practical application of this agent, often in the face of strong opposition, and always under careful, scientific supervision. Resolution No. 1 is: "Tuberculin is a very valuable diagnostic agent, and can yield the greatest assistance in combating tuberculosis. There is no reason for objecting to its general application on the ground that it may aggravate preexisting tubercular lesions." Resolution No. 2 is: "The Congress expresses the desire that government shall order the employment of tuberculin in herds in which the existence of tuberculosis has been established."

The official veterinarians of Germany are advised to use tuberculin, and are supplied with it at a low cost from the government laboratories. A law is now pending in France that will make the use of tuberculin compulsory in all herds in which tuberculosis has been discovered. The infected animals are to be slaughtered; but if they present no clinical evidence of tuberculosis, and the owner wishes to prepare them for slaughter, he is allowed to keep them under quarantine for one year. If upon slaughter it is found that the flesh is unfit for food the owner will receive an indemnity equal to one-half of the value of the condemned meat. If clinical evidence of tuberculosis exists the animal must be slaughtered at once, and the owner receives but one-fourth of the value of the condemned meat.

In Dieckerhoff's *Lehrbuch der Speciellen Pathologie und Therapie für Thieraertze*, published in 1894, and generally recognized as the most reliable work on veterinary practice, this statement occurs: "It is for the owner's interest to have his herd tested with tuberculin in order to discover the suspicious animals, so that they may be separated and placed in another stable and slaughtered as soon as possible. The calves from tuberculous bulls and cows should not be used for breeding.

If these prophylactic measures are not observed it can happen, as has frequently been exemplified, that the value of the herd will be destroyed."

One of the prominent and important steps in all plans for the restriction of bovine tuberculosis that have been proposed by European veterinarians is the compulsory meat-inspection. This furnishes information respecting the location of infected herds, and enables much of the flesh of condemned animals to be saved. While, without a general system of meat-inspection, more time must be devoted to the discovery of infected herds, and the flesh of most of the animals in which tuberculosis is diagnosed must be lost.

So much ground has been covered that I hope I may be allowed in closing to make a brief *résumé* of some points still unsettled in this country on which the veterinary profession in Europe is practically unanimous. This *résumé* is based upon resolutions adopted at representative gatherings of veterinarians during the past two years, or on the *unopposed* publications of distinguished veterinarians.

The system of compulsory meat-inspection should be general. The flesh of tuberculous animals should be subject to special regulations. A part of this flesh must be destroyed, but some of it may safely be used for food.

The milk from cows with tuberculous udders is extremely dangerous. The milk from tuberculous cows with apparently healthy udders may be dangerous, and is always suspicious.

Tuberculin is a reliable diagnostic agent. There need be no fear that tuberculin properly used will cause generalization of preëxisting disease. All tuberculous herds should be tested with tuberculin, and the tuberculous and healthy animals separated.

Since tuberculosis of cattle is of importance in relation to public health as well as of importance in relation to the live-stock industry, it is to the interest of everyone that the disease be restricted as much as possible. Hence when it becomes necessary to condemn tuberculous animals or carcasses the public should share the loss with the owner.

